# Schizophrenia in the context of mental health services in Palestine: a literature review

**DOI:** 10.1186/s13033-020-00375-6

**Published:** 2020-06-15

**Authors:** Mohammad Marie, Zareefa Shaabna, Manahel Saleh

**Affiliations:** 1 Faculty of Medicine and Health Sciences, AN-Najah National University, Nablus, Palestine; 2 Community Mental Health Nursing Program at AN-Najah National University, Nablus, Palestine; 3 Birzeit University, Birzeit, Palestine; 4 Palestine Red Crescent, Tulkarm, Palestine

**Keywords:** Schizophrenia, Schizophrenia in Palestine, Mental health services, Mental health needs

## Abstract

**Background:**

Mental health conditions remain a significant cause of disability in the Arab World. Palestinians are predominantly at a higher risk for mental health problems due to their chronic exposure to political violence, prolonged displacement, and others as limited professional, educational, financial opportunities and mental health services. Schizophrenia is an overwhelming mental illness that affects nearly one percent of the various populations throughout the world. Studies have shown patients with schizophrenia die prematurely and have lower life expectancy compared to the general population. Moreover, antipsychotic medications and client’s lifestyle play a significant role in increased morbidity and mortality in these patients. The present study willingly undertakes a literature review on schizophrenia in the context of mental health services in Palestine.

**Methods:**

Studies were identified through PubMed, Science Direct, Google Scholar, CINAHL, Semantic Scholar and Elsevier.

**Results:**

Twenty-four studies were included in this review; 11 articles related to schizophrenia and thirteen articles related to mental health services in Westbank and Gaza. Results revealed the life of patients with schizophrenia in Palestine is complicated. Barriers as lacking awareness about mental illness, stigma, inconsistent availability of medications, absence of multidisciplinary teamwork, insufficient specialists, fragmented mental health system, occupation, and other obstacles stand in the face of improving the quality of life among these patients.

**Limitations:**

Palestine is a state that is seeking independence with a scarcity of resources. It has been described as “uncharted territories’’ due to a lack of data, resources and records. As a result, there is insufficient data regarding schizophrenia in Palestine. Therefore, a thesis study that estimated 10 years’ risk of coronary heart diseases in patients with schizophrenia was included.

**Conclusions:**

Recommendations include ending the occupation as the leading cause of mental illness for Palestinians and implementing efficient and effective mental health nursing care through the multidisciplinary work and raising awareness regarding mental illness to fight the stigma.

## Background

According to the Palestinian health information center PHIC 2018, the overall population of Palestine is 5 million, divided between the three areas of the West Bank, Gaza and East Jerusalem within the occupied Palestinian territory. The history of Palestine is known by conflict, and the challenging political context has exerted effects on the Palestinian physical, mental wellbeing and lifestyle. It all started in 1948 when the Palestinians considered the War between Arabic countries and Israel as the beginning of the ‘Catastrophe,’ which is known in the Arabic language as ‘Nakba’ during which thousands of Palestinians were forced to leave their homes and became refugees in Gaza, West Bank, and the surrounding countries. Since 1967, the Israeli occupation has negatively impacted the quality of life among Palestinians through imposing poverty, unemployment, ongoing violence and restriction of resources such as water, building materials, electricity, in addition to the intermittent restrictions of movement [1].

The health care system in Palestine is still in its evolutional stage and facing specific challenges linked with occupation and political conflict [2, 3]. The health care system is complex, fragmented, and the essential public health along with the primary care is proposed by four main facilities: The Palestinian Authority (Governmental), the United Nations, NGOs, and the private health care services [3, 4]. According to the World Health Organization (WHO) and the Ministry of Health (MOH), the community mental health was not considered as a priority on the MOH list of budget [5]. The budget for the mental health services consisted of only 2% of the total budget of the MOH. The WHO cooperation with the MOH has been trying to develop the health care system in Palestine, and they have implemented a plan to improve the mental health system in Palestine, including West Bank [6]. As a result, the WHO built a new community mental health centers in each city and has also helped in training numbers of mental health professional teams; although, the total number of mental health nurses continues to remain very few [7]. As this is vital, nurses remain the key to the development of health systems around the world where they stay globally, the largest of the professional groups involved in health care delivery [6].

Nowadays, mental health services in the West Bank and East Jerusalem are community-based care. However, there are only 13 community mental health clinics or centers in West Bank, in addition to one psychiatric hospital in Bethlehem. Mental health services in Palestine continue to be underreported, under-resourced, under-researched, under-supported, and the mental health services are still underfunded; these services are not able to meet the burden of need [7, 8]. Besides, severe lacking in human infrastructural resources, for instance, the total number of the physiatrists are only 20 in the West Bank [3]. Each community mental center contains mostly a psychiatrist, psychologist, or social worker in addition to only one, not well-trained individual or mental health nurse [9]. One of the reasons contributed to the lack of community mental health nurses is the stigma related to nurses who work in community mental health care centers [7]. Moreover, the mental health system has been affected adversely by the political conflict [7]. Restrictions on freedom and movement practiced by Israel considerably limit patients from receiving care outside of their area of residence. The cost of treatment and inconsistent availability of medications on the WHO essential medicines list present further access issues [3]. This is believed to increase the challenges that face the health workers in their daily routines.

With all the challenges that faced the Palestinian people, ‘Sumud’ and health-related Resilience which is in the deeper roots of the Palestinian context play an essential role in remaining steadfast in the face of their daily challenges [10]. However, this does not eliminate that Palestinians can expose to mental disorders like any other population. In fact, with their full history of conflicts, struggle, and poverty, it’s most likely that the number of mental disorders is willing to increase. According to Afana et al. [11], the historical events have made approximately one-third of Palestinians in need of mental health interventions, which causes mental illness one of the largest but least acknowledged of all health problems.

Schizophrenia is a mental disorder that affects approximately one percent of the various populations throughout the world. Schizophrenia is characterized by delusions, hallucinations, disorganized speech, behavior, and other symptoms that cause social or occupational dysfunction. For the diagnosis, symptoms must have been present for at least 6 months and include at least 1 month of active symptoms (American Psychiatric Association 2018). The DSM-5 raises the symptom threshold, requiring that an individual exhibit at least two of the specified symptoms. Patients with schizophrenia are reported to have a shorter life span compared to the general population. A shortened life span could be to the increased frequency of some physical illnesses, particularly diabetes and coronary heart diseases [12, 13].

Quality of life is a holistic view of health from a bio-psycho-social viewpoint, which emerged during the post-World War II period, to enhance the post-war economic wealth and standards of living. Quality of life is strongly associated with diseases. Researchers have observed that national deficiencies that are associated with some mental disorders [14]. Patients with schizophrenia reported making significantly weaker dietary choices, perform less exercise and smoke heavily than the general population [15]. In psychiatric practice, weight gain is a long-recognized and commonly encountered problem [16]. Monitoring the body weight in the early treatment will help to predict those at higher risk for substantial weight gain. Lifestyle therapies and other non-pharmacological interventions were also showed to be effective in controlled clinical trials [17]. Therefore, this literature review sought to highlight schizophrenia in the context of mental health services in Palestine.

## Methodology

### Literature search

The search for the literature was through the following electronic databases: PubMed, Science Direct, Google Scholar, CINAHL, Semantic Scholar and Elsevier from August to November 2019. The following keywords were used: ‘schizophrenia Palestine,’ schizophrenia *West bank, schizophrenia *Gaza, mental health AND Palestine, Palestine AND mental challenges, Arab Mental health AND needs. These keywords were additionally used to search in the Arabic language. A total of 3062 articles was found using the search strategy. 361 publications remained after duplicates, irrelevant and excluded articles were removed. Other papers, which did not appear while searching in the electronic database were obtained via examination of reference lists of published papers. After reviewing in depth of these publications and obtaining necessary data by contacting the authors. 24 studies (24 articles) met the inclusion criteria; eleven articles discussed schizophrenia and thirteen articles related to mental health in Palestine, West Bank and/or Gaza including a one thesis study. All reviewers independently charted the data and discussed the results, and all discrepancies were resolved by the principal investigator (ZS). The studies were grouped by the topic they investigated, and for the critical analysis of empirical articles, the following aspects were considered: study type, the survey instrument used, aim, sample, and the key findings.

### Inclusion and exclusion criteria

Studies were eligible for inclusion if they satisfied the following criteria: (1) Reviewed schizophrenia in Palestine, West Bank and/or Gaza. (2) Reviewed mental health or Mental health services in Palestine, West Bank and/or Gaza. (3) Reviewed Schizophrenia and Mental health services in Palestine, West Bank and/or Gaza. All articles that discussed schizophrenia from a genetic view were excluded.

### Quality assessment

The quality assessment of each study was assessed according to a checklist. The checklist consists of the following items: including clear study aims, representative sample, response rate reported with losses given, an adequate description of data, explicit inclusion and exclusion criteria, valid and reliable measure of mental health and appropriate statistical analysis. The three investigators assessed article quality, and inconsistencies were resolved by the principal investigator (ZS).

### An overview of schizophrenia in the Arabic and the surrounding countries

Mental health conditions remain a significant cause of disability in the Arab World [18]. In Islam, “no responsibility was attributed to a child, a psychotic adult or a sleeping or stuporous person.” The care of people with mental illness under Islam is considered a family responsibility [19–23]. In Arabic culture, such an illness is viewed as a family issue. Whether the person is hospitalized or not or kept in or discharged from the hospital depends not on the individual needs but the desire of the family. Therefore, in Arab culture, the issues of patient consent, autonomy, and decision making are considered family-centered [23]. In the Arab world, families of patients with schizophrenia suffer from stigmatization [24]. It’s a widespread belief in the Arab society that mental illnesses have a devilish and sinful component [25]. Along with stigma, it acts as a barrier in seeking treatment.

The Saudi Arabian Ministry of Health reported that 22.4% of the outpatient mental health services suffer from mental and behavioral disorders caused either by schizophrenia, schizotypal or delusional disorder [26]. According to Foldemo et al. [27], “Quality of life is a complex and multidimensional construct. The majority of definitions include several broad concepts such as well-being, happiness/satisfaction, and achievement of personal goals, social relations, and natural capacity.”. Recently, quality of life was considered an indicator of the impact of diseases on patients who suffer from mental illness [28].

A descriptive qualitative study was conducted at a psychiatric outpatient department in Saudi Arabia in 2010 among 159 patients with schizophrenia to investigate how do patients with schizophrenia perceive their quality of life. Forty-four patients reported that shame of schizophrenia affected their lives negatively, and 39 patients said the embarrassment of having schizophrenia had changed their lives. Patients indicated they would prefer to keep their illness secret for two main reasons: the family shame of having a family member with schizophrenia and the public embarrassment of having schizophrenia. On the other hand, 110 out of 159 patients reported the positive role of religion, such as praying and using the Quran, was positively linked with improving their quality of life [26]. An additional descriptive study was carried out on 160 Jordanian outpatient diagnosed with schizophrenia. The results revealed the patients had a poor quality of life. Age, marital status, education level, stigma against mental illness, and severity of depression were significantly associated with quality of life among Jordanian patients with schizophrenia [29].

Stigma related to mental illness refers to “the view that persons with mental illness are marked, has undesirable characteristics, or deserves reproach because of their mental illness” [30]. Stigmatizing attitudes toward people with mental illness are common. The stigma associated with mental illness brings shame to the family and affect the marriage potential for other siblings, so families keep the illness private and are often reluctant to seek professional help [31]. A comparative study typically aimed to explore the internalized stigma of mental illness among 200 patients with schizophrenia and their families during the follow-up visit in two settings. The 1st clinic attends the outpatients’ clinic for psychiatric patients affiliated to Abbasia hospital, and the 2nd clinic attends the outpatients’ clinic for patients with mental health condition affiliated to Abha psychiatric hospital. By using the stigma impact scale, results revealed that both groups of patients with schizophrenia and their family caregivers possess a high level of the internalizing stigma of mental illness. Results also showed that 80% of family members at Abha hospital agreed that “My life security has been affected by the illness in my family member” and 66% of the family members were strongly agreed that “I feel I have been treated with less respect than usual by others” and “I feel a need to keep my family members illness a secret” [30].

## Results

### Overview of schizophrenia in Palestine

Meeting the need for mental health care for the Palestinian population remains an ongoing struggle [3]. Palestinians are notably at a higher risk for developing mental disorders due to their chronic exposure to political violence, prolonged displacement and insecurity. In addition, the limited professional, educational, and financial opportunities that are linked to the protracted conflicts and instability in the region [32]. These vulnerabilities were compounded by the limited availability of the quality of mental health providers, inconsistent mental health services, and the stigma associated with seeking mental health care [7].

Focusing only on one aspect of the Palestinian reality and gaining more insight into its mental health challenges, especially among patients with schizophrenia. According to the Palestinian Health Information Center (PHIC 2016), the incidence rates for newly reported cases in the West Bank showed that schizophrenia was the third-highest incidence in mental disorders, with it being the highest—Number one—in the treatment with 30,008 cases.

The life and characteristics of patients with schizophrenia seem to be vouge. Studies have investigated the lifestyle and clinical features of patients with schizophrenia in Palestine. A cross-sectional study design conducted at the governmental primary psychiatric health care centers in Northern West Bank and implemented a survey to investigate the different lifestyle parameters, diet, body mass index, smoking, and unemployment among 250 patients with schizophrenia in Palestine. Results showed that 43.6% had completed their elementary level of education; 41.6% with a high school level, 14.8% with a 2-year diploma, and none of the clients obtained a bachelor’s degree. One hundred and ninety-seven (78.80%) participants were without a job, and the number of working patients was only 53 (21.2%). Moreover, only 82 clients (32.8% from the total number of patients) had an average BMI values most of them were males (60 males and 13 females), and the number of patients with schizophrenia suffering from overweight and obesity was high (67.2%), and the average of waist circumference for most of the clients was abnormal (97.8 ± 13.4). In addition to the previous, over half of the participants were smokers representing (61.20%) [33].

Another study investigated the clinical characteristics of schizophrenia among three different group categories (Negev Bedouin, Galilee Palestinians and Palestinian Authority). The results revealed that from the total 50 patients in the Palestinian category; (78%) of the patients were males (66%) were single/divorced (70%) were unemployed and (70%) had low to medium education level. Moreover, somatic delusions were the highest delusions in this category representing (86%), followed by Persecution delusions representing (82%), and Jealousy delusions were the lowest among Palestinian Authority patients (4%). Among all of the three different categories, Palestinian authority patients had the most moderate disability insurance coverage compared with the other two groups [34].

Moreover, a cross-sectional study design was carried out at four governmental primary psychiatric health centers using patients’ medical files to investigate schizophrenia treatment guidelines in the care centers located in Nablus, Tukaram, Jenin, and Qalqilya. Both newly diagnosed patients and patients who were not on antipsychotic therapy were excluded. The characteristics of the 250 participants in the study were 182; (73.8%) male patients, 145 (58%) live in village/camp, 213 (85.2%) have completed school education or less, 112 (44.8%) were single/divorced, 153 (61.2%) were smokers, 219 (87.6%) without a job and 161 (64.4%) reported having a duration of illness for more than 10 years [35].

Antipsychotic drug therapy is considered to be one of the treatment regimens for schizophrenia and has been reported to successfully minimize the frequency of acute episodes for schizophrenia and hospitalization [36]. According to Sweileh et al. [35], several major well-known algorithms were used for the treatment of schizophrenia. Schizophrenia treatment guidelines in Palestine were investigated, and results revealed there were 406 prescriptions of antipsychotic drugs used by the 250 patients. The antipsychotics were primarily from First-generation type (FGT) (85.7%), and the most common antipsychotic medications that were consumed by the patients: Chlorpromazine tablet (31.5%), followed by Fluphenazine IM depot injection (30.8%), Haloperidol tablet (18.2%), Clozapine (8.6%), Olanzapine (3.7%), Haloperidol Decanoate (2.7%), Risperidone (2%), Trifluoperazine (1.7%), Thioridazine (0.2%) and Zuclopenthixol (0.5%). This study also indicated that antipsychotic prescribing was not in the conformance with the international guidelines with respect to maintenance dose and combination therapy; categorization of Chlorpromazine dose equivalencies (CPZeq) showed that 88 (35.2%) clients were using sub-therapeutic treatments (< 300 mg CPZeq), 105 (42%) were using the optimum dose (300–600 mg CPZeq), 57 (22.8%) were using supratherapeutic treatments (> 600 mg CPZeq) and 7 (2.8%) were using the supra-maximal dose (CPZeq > 1000 mg) [35].

Antipsychotic medication adherence and satisfaction were also assessed in patients with schizophrenia. A cross-sectional study was conducted in 2010 at Al-Makhfya psychiatric health center in Nablus. Medication adherence was assessed using the 8-item Morisky Medication Adherence Scale (MMAS-8), treatment satisfaction was assessed using the Treatment Satisfaction Questionnaire for Medication (TSQM 1.4), and psychiatric symptoms were evaluated using the expanded Brief Psychiatric Rating Scale (BPRS-E). Results revealed medication nonadherence was common and was associated with low treatment satisfaction scores and poor psychiatric scores. In addition, the majority of patients with schizophrenia were nonadherent, and the younger people had significantly lower adherence scores than the elderly (P = 0.028) [25].

Antipsychotic medications cause serious side effects, including metabolic syndrome (MS) [37]. Metabolic syndrome is defined as a cluster of conditions that occur simultaneously, which increases the risk of developing heart diseases, stroke, and type 2 diabetes. These conditions included elevated blood pressure, high blood sugar, excess body fat around the waist, and abnormal cholesterol or triglyceride levels (Adult Treatment Panel III 2004). A cross-sectional study conducted from August 2011 until February 2012 at governmental primary healthcare psychiatric centers in Northern West-Bank, investigated the prevalence of metabolic syndrome (MS) among 250 patients with schizophrenia above the age of 16 and were diagnosed according to DSM IV. Using the Adult Treatment Panel III (ATP III) criteria, results revealed that 109 (43.6%) of patients met the criteria for the syndrome; 39% in males and 55.9% in female patients. Among males, elevated levels of triglyceride were the most common metabolic component compared to females who have abdominal obesity as a common metabolic component. Elevated fasting blood sugar was the least common metabolic dysregulation in both genders. This study also revealed by using the univariate analysis that MS was significantly higher with older age, female gender, longer duration of illness, abdominal obesity, smoking, higher systolic and diastolic blood pressure, high triglycerides, low HDL-C, and fasting plasma glucose compared to the multiple logistic regression analysis which showed that only systolic blood pressure, high triglycerides, high fasting plasma glucose and low HDL-C were significant predictors of MS in these patients. This study also supported the previous studies in the characteristics of patients with schizophrenia; 213 (85.2%) had only school education or less, 122 (44.8%) were single or divorced, 153 (61.2%) were smokers and 219 (87.6%) without a job [13].

Metabolic syndrome is not the only complication that affects this category of patients. Diabetes, anemia, cardiovascular diseases, and more were also studied in these patients. A cross-sectional study was carried out in four governmental primary psychiatric healthcare centers in Northern West-Bank from August 2011 until February 2012 and used a survey to examine the prevalence of Diabetes Mellitus among 250 patients with schizophrenia. The criteria for the patients were: age above 16 years old, diagnosed with schizophrenia as defined by DSM IV, didn’t suffer from an acute attack of illness during the past year and their drug regimen had been unchanged in the last 6 months. Results revealed that among the study sample; 189 (75.6%) were considered to have euglycemia, and 61 (24.4%) have dysglycemia (defined as FBG ≥ 110 mg/dL). Based on the WHO criteria, 27 patients (10.8%) had Diabetes and 34 (13.6%) had prediabetes. Results of multiple logistic regressions showed that only advancing age and abnormal waist circumference were significant predicators of dysglycemia among patients with schizophrenia with a significant (P = 0.003) and (P = 0.013) respectively [12].

Inadequate or inappropriate dietary habits increase the risk of anemia in patients with schizophrenia [38]. Many studies have demonstrated that patients with schizophrenia make poor nutritional choices [39]. A cross-sectional study was conducted between August 2011 and February 2012, covering four governmental primary psychiatric health care centers located throughout the Northern West Bank to report the prevalence of anemia among 250 patients with schizophrenia. Results revealed the number of anemic females was 38 (55.9%) out of 68 patients, while the number of anemic males was 25 (13.7%) out of 182 patients (*P* value < 0.01). In comparison, 6.1% of males and 11.8% of female patients had leucopenia, 7.7% of males and 7.3% of females had leukocytosis, 5.5% of males and 4.4% of females had thrombocytopenia and 1.1% of males and 5.9% of females had thrombocytosis. Results revealed an unhealthy lifestyle and poor dietary choices represent the primary cause of anemia among these patients [38].

Coronary heart diseases and mental illness are one of the leading causes of morbidity and mortality worldwide. Research has revealed several, and sometimes surprising links between both CHD and mental illness and has even suggested that both may actually cause one another [40]. A cross-sectional study design was carried at four governmental primary psychiatric health care centers in northern West-Bank to estimate the 10 years’ risk of coronary heart diseases (CHD) among 112 patients with schizophrenia. Results revealed one-fifth of the patients had a CHD risk of 10% [41].

Globally, approximately 3% of the total burden of human diseases is attributable to schizophrenia [42]. The WHO has estimated that around 40–90% of patients having schizophrenia live with their families [43]. A cross-sectional study conducted at the Gaza governmental community mental health centers aimed to investigate the burden of care experienced by 120 caregivers of patients with schizophrenia. Results revealed the sociodemographic characteristics of patients were the following: the majority of the sample were male patients representing 62.5%, and about half of the sample were married 53.3%, 28.3% were single, 16.7% divorced, and 1.7% widowed. The educational level showed that 10% were illiterate, and 40.9% completed their primary education, 29.2% completed secondary school, 5.8% had a diploma, 13.3% had a bachelor’s degree, and 0.8% had a master’s degree or higher. The rate of unemployment was 87.5%, and regarding the medical income, 81.7% had less than 1000 NIS, 13.3% had 1000 to 2500 NIS, while only 5% had a monthly salary of 2500 NIS or more. The burden on caregivers for patients with schizophrenia was measured using the burden assessment scale. Results revealed that caregivers suffered from a high level of a total burden representing 74.5% and the distribution was as the following: physical 81%, financial 79.3%, psychological 72.4%, and social burden 68.3%. Results also revealed that there were significant differences in the level of responsibility, education, occupation, and monthly income for both caregivers and patients [44].

Stigma among psychiatric patients is dangerous as it interferes with the understanding, gaining support from friends and family, delays getting help, and self-blame [45]. A descriptive study was conducted at the outpatient clinics of the only psychiatric hospital in the Gaza Strip and used a questionnaire to assess the impact of stigma on the daily life of 106 psychiatric patients. Results revealed the majority of the patients were males representing 61.3%, 50% of patients were single, and stigma had a significant effect on the daily life of patients with mental illness. The patients highest reports were as the following: “I fell shy because of my psychiatric illness, and this prevents me from expressing my point of view easily” (P = 0.004), “I prefer giving a pen name and change my look and clothes when I go to the psychiatrist to avoid an embarrassment” (P = 0.007), and then “My request was rejected for several jobs because of my psychiatric illness” (P < 0.001) [46].

## Discussion

The following discussion will be divided into two theme categories discussing both patients and mental health care providers in the context of mental health services.

### Patients with schizophrenia in the context of mental health services

Psychotic disorders are connected with unhealthy life habits, such as poor diet, smoking, and physical inactivity [47]. Schizophrenia is a disabling psychiatric disorder due to the disease itself, medications, and lifestyle-related factors [48]. Therefore, patients with schizophrenia are a suitable target group for health promotion and interventions. From the previously presented studies in Palestine, it was clear that patients with schizophrenia suffered from a seriously unhealthy lifestyle and health challenges during their lifetime. The high rates of obesity and anemia among patients with schizophrenia in the previously presented studies may be attributed to that these patients usually have inadequate nutritional intake. Research has shown that patients with schizophrenia drink more carbonated drinks, but fewer consume milk, fish, nuts and vegetables [39]. Moreover, these patients also tend to perform only limited amounts of exercise; factors such as illness, sedative medication, and lack of motivation may be relevant [15, 49]. Besides, over half of the previous study samples were smokers. Among the mentally ill, smoking prevalence is the highest in clients with schizophrenia representing 80%, whereas it is 20% in the general population [50, 51]. Studies have also indicated a strong association between schizophrenia and smoking but with no ultimate explanation for this prevalence [52].

In the previously presented studies, most of their samples involved male patients. Although schizophrenia affects men and women with equal frequency, the illness is expressed differently between both sexes. Women tend to have better premorbid functioning, a later age at onset, a distinct symptom profile and better course of illness. These gender differences are thought to arise from the interplay between hormonal and psychosocial factors [53]. Results also suggested that the high ratio of schizophrenia among male patients in Palestine may be due to the economic crises and political unrest especially that Israel has detained approximately 40% of men in the occupied Palestinian territory, often for indeterminate periods for no specific charges and often suffering mistreatment or outright torture while arrested [54]. Moreover, these results may also explain the reasons behind why persecution delusions represent the most prominent delusions among Palestinian authority patients. Persecutory delusions occur when someone believes others are out to harm them. It’s a type of paranoid thinking that can be part of several different mental illnesses [55].

It’s firmly established that patients with schizophrenia experience markedly high rates of unemployment due to difficulties in social and cognitive function, self-care, residual negative symptoms and social exclusion [56]. Research also indicates that patients with schizophrenia have a lower pre-morbid IQ [57]. This may explain the reasons why education in the previous studies was confined merely to primary education. However, this does not eliminate this category of patients has no development capabilities. Occupational therapy and rehabilitation were found, along with used medication, to improve the symptoms of schizophrenia [56]. However, in Palestine there is a near absence of occupational and rehabilitation plans in community mental health services due to conspicuous lack of training and understaffed mental health nurses [58].

Stigma related to mental illness is prevalent, and this may prevent or delay patients from presenting [59]. The internalized stigma that accompanies patients to devalue themselves and increase family concerns about social standing or marriage prospects for other siblings, especially if the patient was female act as a barrier for families not to seek mental health services. This reason may also justify why the majority of the samples in the previous studies were male patients. Stigma related to mental illness carries a crucial role in increasing the amount of unemployment. In one specific study, many patients reported, “My request was rejected for several jobs because of my psychiatric illness” [45]. The absence of legislation supporting the right of mentally ill patients to obtain a job in Palestine exaggerates the unemployment rate among patients with schizophrenia. The unemployment rates are 20% and 31% in the West Bank and Gaza respectively, and the median family size in the West Bank is 5.4 with an average income per adult 9 USD/day [60].

Furthermore, patients experience numerous barriers to care. One significant barrier is awareness [3, 7]. So far, many Palestinians haven’t been aware of mental health issues, and how they present, behaviors associated with depressions, and other common illnesses are often not understood to be psychiatric problems. For instance, in one survey of mothers in Gaza, only 19.6% perceived suicidal behavior as a manifestation of mental health problems [61]. Patients may be labeled pejoratively, viewed as lazy or crazy, but there is not a widespread understanding that they suffer from a medical condition.

First-generation antipsychotics (FGAs) are associated with a range of adverse effects that can significantly reduce patients’ quality of life and contribute to nonadherence [62]. This confirms the results of the previously presented study that revealed medication nonadherence among the patients with a significant (P value = 0.028). Besides, the cost of treatment, medications, and inconsistent availability of medications on the WHO essential medications list present additional access issues [3]. Even at least 80% of essential psychotropic medications are provided free of charge, whenever a shortage in these medications happens, the cost of private purchases of antipsychotic and antidepressant medication is 5% and 7% of the minimum daily wage in Gaza, respectively [63]. Switching between the drugs when inconstant availability exaggerates the adherence and satisfaction. Furthermore, focusing merely on medication prescription without combining with psychological treatment such as cognitive behavioral therapy, psychoeducation, and family therapy complicates the life of these patients.

Metabolic syndrome, diabetes, anemia, CVD and other medical complications stand in the face of improving the quality of life in patients with schizophrenia. The absence of the multidisciplinary teamwork and collaboration between the health care providers to screen and follow up the patient’s test results place the patients’ health in danger. Lacking multidisciplinary working was evident while investigating schizophrenia treatment guidelines. Results revealed 35.2% of patients were using sub-therapeutic treatments, 22.8% were using supratherapeutic therapies and 2.8% were using supra-maximal doses. Furthermore, the results shown in metabolic syndrome, anemia, and CVD confirm there is no screening or follow up plans for these patients. The style of care should move toward multidisciplinary working based on a “non-hierarchical” mental health system, and families should be integrated into the heart of care [7].

### Mental health care providers in the context of mental health services

A critical barrier to care is the shortage of health care providers specialized in psychiatric, mental health and psychology. Published data counts only 20 Palestinian psychiatrists in West Bank and Gaza combined [3, 64]. There are only a few doctoral levels of psychologists and the training in the programs offering bachelor’s and master’s degree in psychology and social work lack sustainable clinical exposure [3]. The total number of nurses who work in community mental health workplaces in the West Bank is only 17 [65]. These nurses who work in the clinics/centers have been unable to provide mental health care accurately. Some of them work as receptionists or clerks due to the severe shortage of employees or lack of training [7]. In a particular study, the findings revealed that none of the nurses who work in mental health services had a master’s degree in nursing mental health [58].

Another barrier to develop the skills of mental health care providers is the lack of supervision. A report has mentioned that more than 50 Palestinian therapists have been trained on CBT techniques. However, the deficit in supervision for trainees following their initial CBT training acts as a barrier to care. Without this adequate follow-up, trainees are less likely to maintain their new skills properly and adequately incorporate them into future practice. This deficit instantly arises from multiple sources, including limited experience in Palestine with the practice of personal supervision, the lack of understanding among program administrators about the cost-effectiveness of supervision and the resentment therapists may feel towards CBT as an unwelcome modern technique that has not previously been a part of their work [66].

The absence of multidisciplinary teamwork in providing care presents a further challenge. Physicians have frequently refused to involve nurses in assessment, evaluation, and treatment strategy effectively [58]. Besides, stigma related to nurses who work in mental health services is also present. According to Manasra, mental health nursing is described as not being a desired job due to the associated stigma [67]. For instance, a nurse reported, “a nurse in the primary health department, ridiculed and laughed at me, said that I am crazy to work here in the clinic. For your information, when this job was offered to several nurses, no one accepted it but me” [58]. Other barriers to nurses who work in mental health services are the inconsistency of care services delivery, including health care supplies, medications and salaries. For example, a nurse was unable to administer the intramuscular injection due to the unavailability of syringes and needles in the center for 2 months [58]. Moreover, the inconsistent availability of medications in the Ministry of health, which their service depends on Israel’s occupation in allowing the delivery of the medical supplies’ places additional challenges on mental health nurses in dealing with nonadherence, relapses, and negative symptoms [58]. Moreover, occupation plays an essential role in causing mental illness. The historical events have made around one-third of the Palestinians in need of mental health interventions [11]. Occupation has also played an essential role in confiscating financial support, international aid, and movement restrictions. Additionally, imposing poverty, unemployment, violence, trauma, and limitation of necessary resources like water, building materials and electricity [1, 7, 68].

## Conclusion

In conclusion, the life of patients with schizophrenia in the Arabic world and particularly in Palestine, is complicated. Barriers as the presence of occupation, lacking awareness about mental illness, stigma, inconsistent availability of medications, absence of multidisciplinary teamwork, insufficient specialists, fragmented mental health system, and others stand in the face of improving the quality of life in schizophrenia patients. The priority of the Palestinian health care system should be toward improving the quality and increasing the number of qualified mental health providers, especially mental health nursing, and stop the occupation as the primary and only prevention.

Recommendations as increasing awareness about mental health and anti-stigma campaigns should be considered. Moreover, efficient and effective care through multidisciplinary teamwork should be implemented. Furthermore, increasing, empowering, and training mental health nurses on psychotherapies to enhance the quality of care, and allowing mental health nurses to prescribe specific monthly medications independently as in the UK to reduce the burden on the limited number of psychiatrists.

## Limitations

The literature review has discussed schizophrenia in the context of mental health services in Palestine. Palestine is a state that is seeking independence with a scarcity of resources. Palestine has been described as “uncharted territories’’ due to a lack of data, resources and records. As a result, there is insufficient data regarding schizophrenia in Palestine. Therefore, a thesis study that estimated 10 years’ risk of coronary heart diseases in patients with schizophrenia was included.

## Data Availability

This is an evidence synthesis study, all data is available from the primary research studies, or can be circulated from the corresponding author.
